# Spontaneous Rupture of a Hydronephrotic Kidney Secondary to Congenital Ureteropelvic Junction Obstruction: A Report of a Rare Case

**DOI:** 10.7759/cureus.89610

**Published:** 2025-08-08

**Authors:** Lakshay Singla, Paramjit S Kahlon, Prem Chand

**Affiliations:** 1 General Surgery, Rajindra Hospital, Government Medical College, Patiala, IND; 2 General Surgery, Government Medical College, Patiala, IND; 3 General Surgery, Government Medical College, patiala, IND

**Keywords:** hydronephrosis, open pyeloplasty, pelvi-ureteric junction obstruction, perinephric collections, spontaneous renal rupture

## Abstract

Spontaneous rupture of the pelvicalyceal system due to obstructive uropathy is a rare urological emergency. It can mimic other abdominal catastrophes and may be underrecognized, especially in young patients. We report the case of a 17-year-old male presenting with sudden-onset left flank pain, fever, and vomiting. Imaging revealed a grossly dilated left pelvicalyceal system with contrast extravasation into the perinephric space, consistent with spontaneous rupture secondary to ureteropelvic junction (UPJ) obstruction. The patient was managed surgically via open Anderson-Hynes pyeloplasty with successful resolution. This case highlights the importance of early recognition and surgical management of spontaneous renal rupture in the context of UPJ obstruction. High clinical suspicion, timely imaging, and prompt intervention are crucial to prevent morbidity.

## Introduction

Uretero-pelvic junction (UPJ) obstruction is the leading cause of hydronephrosis in children [1], with an estimated incidence of one in every 1,000 to 2,000 newborns, yet spontaneous rupture secondary to UPJ obstruction is scarcely reported in the literature. Spontaneous rupture of the upper urinary tract is an uncommon but potentially serious complication of obstructive uropathy, most often occurring in the setting of UPJ obstruction. It is characterized by a sudden rise in intrapelvic pressure leading to forniceal rupture and extravasation of urine or contrast into the perinephric or retroperitoneal space [2]. Although this phenomenon is well-documented in adults, its occurrence in adolescents is rare and frequently underdiagnosed due to nonspecific symptoms in the form of vague or atypical localized abdominal pain, and lower clinical suspicion [3] for congenital anomalies like UPJ obstruction in this age group can contribute to delayed recognition.

Patients typically present with acute flank or abdominal pain, fever, and signs of systemic inflammation. Imaging, particularly contrast-enhanced computed tomography (CECT), plays a pivotal role in diagnosis, revealing a dilated pelvicalyceal system with evidence of urinary extravasation [4]. If untreated, complications such as urinoma, perinephric abscess, or permanent renal damage may occur [5].

We present a rare case of spontaneous rupture of hydronephrosis in a 17-year-old male secondary to UPJ obstruction, managed successfully with open Anderson-Hynes pyeloplasty. Due to the absence of trauma, stone disease, or prior interventions, and its occurrence in an adolescent male, an age group where congenital anomalies like UPJ obstruction are less commonly suspected, this case report is unique and thus contributes to the limited body of evidence and underscores the need for high clinical suspicion in similar scenarios. This case highlights the need for early recognition and surgical intervention in such presentations.

## Case presentation

A 17-year-old male presented with an acute onset of severe left flank pain, accompanied by fever and episodes of vomiting for one day. There was no preceding history of trauma, hematuria, urinary tract infection, or prior urological complaints. On clinical examination, the patient was febrile and exhibited tenderness in the left costovertebral angle. Mild abdominal distension and flank guarding were noted. Initial laboratory investigations (Table 1) revealed leukocytosis and elevated C-reactive protein, while renal function parameters remained within normal limits. Urinalysis demonstrated sterile pyuria.

**Table 1:** Laboratory results Leukocytosis and elevated C-reactive protein indicated systemic inflammation. Renal function tests were within normal limits, and urinalysis revealed sterile pyuria.

Investigation	Result	Reference range
Total leucocyte count	15000	4000-11000 cu/mm
C-reactive protein	280	<5 mg/L
Serum creatinine	1.1	0.6–1.2 mg/dL
Blood urea nitrogen	15	7–20 mg/dL
Urinalysis	Sterile pyuria	No bacteria or WBCs

**Figure 1:**
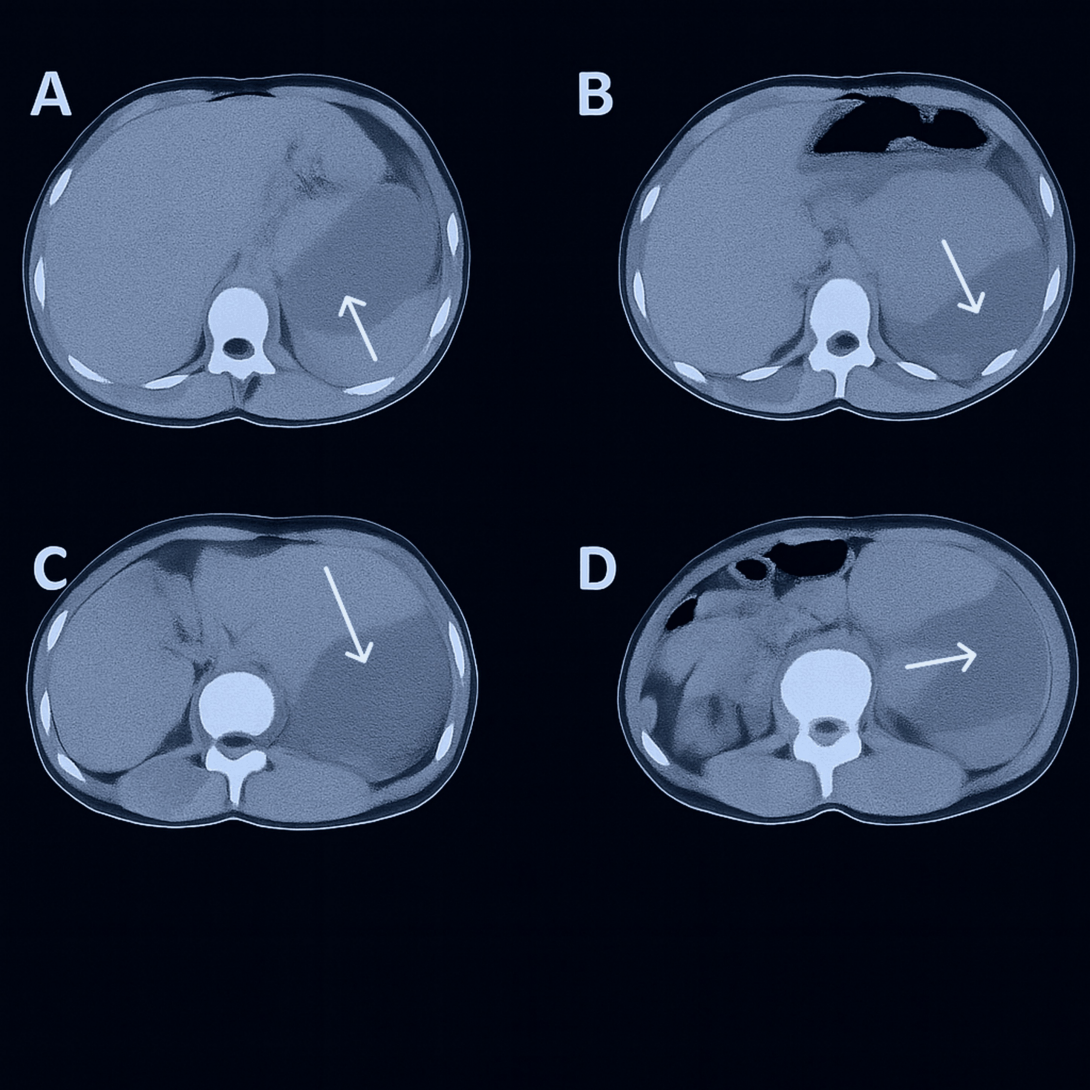
CT urogram axial view Panels A-D: Sequential axial cuts demonstrate significant left hydronephrosis with perinephric fluid collection and contrast extravasation (indicated by arrows) consistent with spontaneous rupture of the renal collecting system. These findings were critical in confirming the diagnosis and guiding surgical management.

**Figure 2:**
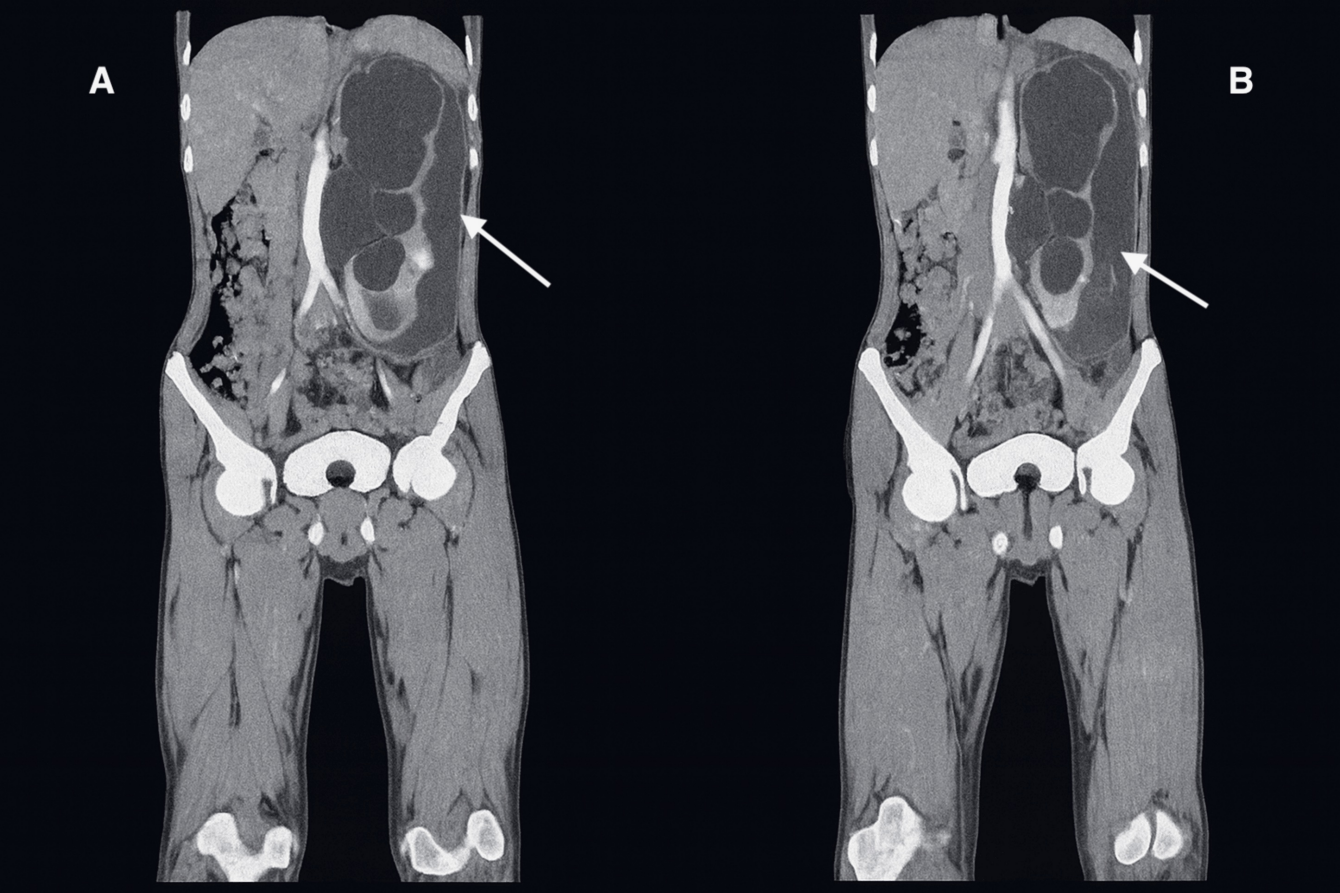
CT urogram coronal view Coronal CT images show the spontaneous rupture of a hydronephrotic kidney. A: Markedly dilated pelvicalyceal system with thinning of renal parenchyma; B: Perinephric fluid collection consistent with urinary extravasation. These findings are consistent with spontaneous pelvicalyceal rupture secondary to high-pressure obstruction.

Ultrasonography of the abdomen (image unavailable) revealed gross left-sided hydronephrosis with thinning of the renal cortex and associated anechoic perinephric fluid collection, consistent with a high-grade obstruction and possible urinary extravasation. The CECT of the abdomen demonstrated gross dilatation of the left pelvicalyceal system with contrast extravasation into the perinephric space, consistent with spontaneous rupture secondary to UPJ obstruction (Figures 1-2). A preoperative nuclear study was not performed in view of the emergency presentation. 

The patient was initially stabilized with intravenous fluids, broad-spectrum antibiotics, and analgesia. Given the persistence of symptoms and imaging findings, definitive surgical intervention was undertaken. Intraoperatively, the left kidney was found to be grossly hydronephrotic with a narrowed ureteropelvic junction, consistent with high-grade hydronephrosis and congenital obstruction with a markedly dilated renal pelvis with thinned overlying parenchyma and a stenotic UPJ, without evidence of intrinsic lesions or aberrant crossing vessels. An open Anderson-Hynes dismembered pyeloplasty was performed to correct the UPJ obstruction. Intraoperatively, a double-J (DJ) ureteric stent was inserted to maintain ureteral patency, facilitate the drainage of urine, and prevent postoperative complications such as urinary leakage or obstruction at the anastomotic site. The stent also served to support the healing process during the early postoperative period.

The postoperative course was uneventful. The perinephric drain was removed on the third postoperative day, and the patient was discharged on the fifth postoperative day with a DJ ureteric stent in situ. The stent was removed at six weeks. While formal postoperative imaging was not digitally archived, clinical follow-up indicated symptomatic improvement. An ultrasound performed six weeks postoperatively showed resolution of the hydronephrosis with no residual perinephric collection.

At the three-month follow-up, the patient remained asymptomatic, with preserved renal function and no radiological evidence of residual obstruction or perinephric collection. Thus, key diagnostic clues for spontaneous rupture include acute flank pain without a history of trauma or urolithiasis, sterile pyuria, and the presence of perinephric fluid collection on imaging. These findings help in prompt diagnosis. 

## Discussion

Spontaneous rupture of the renal collecting system is a rare clinical entity and often presents a diagnostic dilemma due to its nonspecific symptoms. While most commonly associated with obstructive uropathy, other causes include urolithiasis, external trauma, malignancy, and iatrogenic injury [6]. Among obstructive etiologies, UPJ obstruction accounts for a small subset of cases, especially in the pediatric and adolescent population.

The mechanism of rupture is believed to involve a sudden rise in intrapelvic pressure resulting in forniceal rupture. This leads to extravasation of urine into the perinephric or retroperitoneal space, potentially forming a urinoma or leading to perinephric inflammation and fibrosis. In the absence of trauma or infection, such a presentation is often misdiagnosed as other acute abdominal pathologies, delaying definitive management.

Imaging plays a central role in diagnosis. While ultrasonography may reveal hydronephrosis and perinephric collection, CT is the preferred imaging modality for evaluating blunt renal trauma. In contrast, intravenous urography is typically reserved for assessing overall renal function, particularly in hemodynamically unstable patients, as it is particularly useful in evaluating traumatic injuries to the kidneys with pre-existing abnormalities [7]. In our patient, the CT clearly demonstrated extravasation of contrast from the renal pelvis into the perinephric space, confirming the diagnosis.

There is no universally established protocol for managing the spontaneous rupture of hydronephrosis. Treatment strategies may include conservative observation, percutaneous drainage, endoscopic stenting, or surgical interventions such as ureteric reconstruction or, in extreme cases, nephroureterectomy [8,9]. However, in patients with complete obstruction or recurrent symptoms, surgical correction is warranted. In our case, open Anderson-Hynes pyeloplasty was performed due to significant hydronephrosis and persistent symptoms. Surgical repair remains the definitive treatment for UPJ obstruction and has shown excellent long-term outcomes [10].

There are documented cases in the literature where conservative treatment using analgesics and antibiotics has led to favorable outcomes. While nonoperative management is generally advocated for hemodynamically stable patients, some reports suggest that endourological procedures may be required if initial conservative measures prove ineffective [11,12]. Our decision to proceed with open pyeloplasty was guided by the extent of hydronephrosis, persistent symptoms, and concern for long-term renal preservation.

Spontaneous rupture secondary to UPJ obstruction in adolescents is extremely rare, with only isolated reports in the literature. Timely diagnosis and intervention are critical to preserve renal function and prevent complications such as urinoma, abscess, or loss of renal unit. 

## Conclusions

Spontaneous rupture of hydronephrosis secondary to ureteropelvic junction obstruction represents a rare but clinically significant emergency, especially in the adolescent population. Early recognition through cross-sectional imaging and timely surgical intervention are critical in preserving renal function and preventing complications. Pyeloplasty in the setting of spontaneous forniceal rupture appears to yield outcomes comparable to non-ruptured UPJ obstruction, although long-term studies are needed to validate this observation. This case reinforces the importance of considering UPJ obstruction as a differential in young patients presenting with unexplained flank pain and radiological evidence of perinephric collection, even in the absence of trauma or calculi.

## References

[1] (2024). Pediatric ureteropelvic junction obstruction: practice essentials, anatomy, pathophysiology. eMedicine [Internet.

[2] Tylski M, Muras-Szwedziak K, Nowicki M (2021). Idiopathic spontaneous rupture of renal pelvis in a single functioning kidney. Case Rep Nephrol Dial.

[3] Schulman A, Wuilleumier JP, Teper E (2015). Delayed recognition of a ureteropelvic junction obstruction in a young adult female. Case Rep Urol.

[4] Lowe FC, Zagoria RJ (2012). Emergency urology. Campbell-Walsh Urology.

[5] Thotakura R, Anjum F (2023). Hydronephrosis and hydroureter. https://www.ncbi.nlm.nih.gov/books/NBK563217/.

[6] Gulati A, Prakash M, Bhatia A, Mavuduru R, Khandelwal N (2013). Spontaneous rupture of renal pelvis. Am J Emerg Med.

[7] Kawashima A, Sandler CM, Corl FM, West OC, Tamm EP, Fishman EK, Goldman SM (2001). Imaging of renal trauma: a comprehensive review. Radiographics.

[8] Chen GH, Hsiao PJ, Chang YH (2014). Spontaneous ureteral rupture and review of the literature. Am J Emerg Med.

[9] Akpinar H, Kural AR, Tüfek I, Obek C, Demirkesen O, Solok V, Gürtug A (2002). Spontaneous ureteral rupture: is immediate surgical intervention always necessary? Presentation of four cases and review of the literature. J Endourol.

[10] Uhlig A, Uhlig J, Trojan L, Hinterthaner M, von Hammerstein-Equord A, Strauss A (2019). Surgical approaches for treatment of ureteropelvic junction obstruction — a systematic review and network meta-analysis. BMC Urol.

[11] Moore EE, Cogbill TH, Jurkovich GJ (1992). Organ injury scaling. III: chest wall, abdominal vascular, ureter, bladder, and urethra. J Trauma.

[12] Inahara M, Kojima S, Takei K (2009). Two cases of spontaneous rupture of upper urinary tract caused by the primary ureteral or renal pelvic tumor: a case report. Acta Urol Jpn.

